# Supplementary material for: Splice-Junction-Based Mapping of Alternative Isoforms in the Human Proteome
Source: Cell Rep. Author manuscript; Available in PMC 2020 Jan 15. (PMC6961840; doi:10.1016/j.celrep.2019.11.026)
Supplement: 2 [file NIHMS1546469-supplement-2.zip › DF1/PXD010154/Lung/Lung_34_MAP4_MYHDDDLADLVFPSSATADTSIFAGQNDPLKDSYVPLELAK.pdf]

A

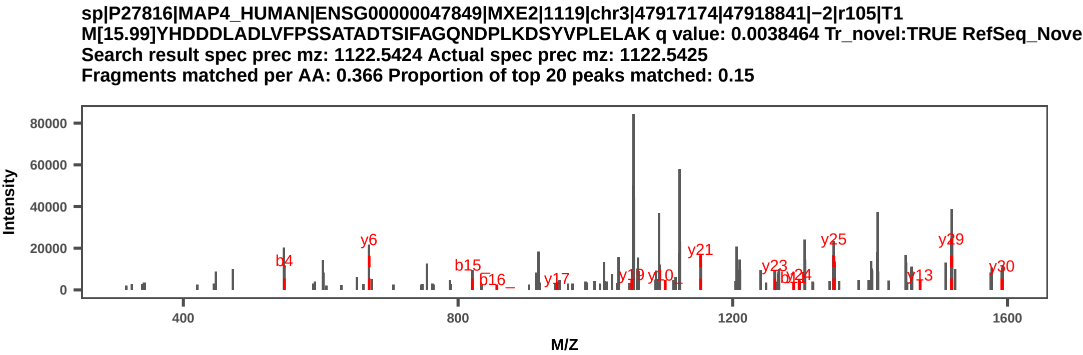
sp|P27816|MAP4_HUMAN|ENSG00000047849|MXE2|1119|chr3|47917174|47918841|−2|r105|T1
M[15.99]YHDDDLADLVFPSSATADTSIFAGQNDPLKDSYVPLELAK q value: 0.0038464 Tr_novel:TRUE RefSeq_Nove
Search result spec prec mz: 1122.5424 Actual spec prec mz: 1122.5425
Fragments matched per AA: 0.366 Proportion of top 20 peaks matched: 0.15
Intensity
M/Z
b4
y6
b15
b16
y17
y19
y10
y21
y23
y24
y25
y13
y29
y30

B

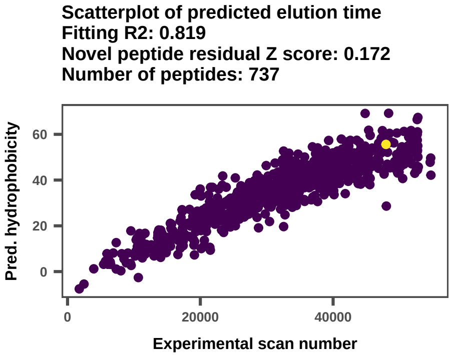
Scatterplot of predicted elution time
Fitting R2: 0.819
Novel peptide residual Z score: 0.172
Number of peptides: 737
Pred. hydrophobicity
Experimental scan number

C

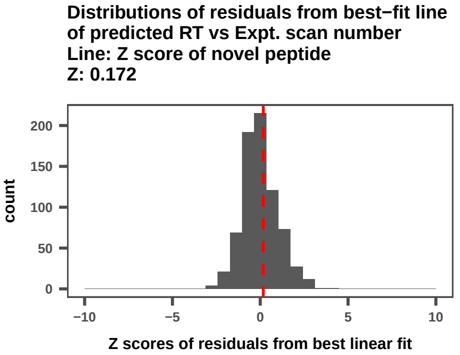
Distributions of residuals from best−fit line
of predicted RT vs Expt. scan number
Line: Z score of novel peptide
Z: 0.172
count
Z scores of residuals from best linear fit
